# Do extreme summers increase blood vitamin D (25-hydroxyvitamin D) levels?

**DOI:** 10.1371/journal.pone.0242230

**Published:** 2020-11-10

**Authors:** Frank Bernhard Kraus, Daniel Medenwald, Beatrice Ludwig-Kraus

**Affiliations:** 1 Central Laboratory, University Hospital Halle, Halle/Saale, Germany; 2 Institute of Medical Epidemiology, Biostatistics and Informatics, Martin-Luther-University Halle-Wittenberg, Halle, Germany; 3 Department of Radiation Oncology, University Hospital Halle (Saale), Halle/Saale, Germany; University of Strathclyde, UNITED KINGDOM

## Abstract

Climate change is expected to increase the frequency of extreme weather events, such as extended heat waves and droughts in the northern hemisphere. Besides affecting ecosystems worldwide, these changes in climate patterns will also affect the environmental health of human populations. While the medical community is mostly concerned with the negative impact of climate change, there might also be some beneficial effects. In this study we used laboratory data from a large university clinic in Germany (n = 13 406), to test for any detectable impact of two extreme summers on Vitamin-D [25(OH)D] plasma concentrations over a six year period (2014–2019). For the two years with extreme summers (2018 and 2019) the 25(OH)D plasma concentrations were significantly higher than in the previous four years (p < 0.001). A time series analysis (autoregressive term, AR, φ = 0.84, with an AR of one indicating a persistent effect) showed that 25(OH)D concentrations rise by 0.04 nmol/l (95% CI: 0.04–0.05 nmol/l) per hour of sunshine. The incidence of vitamin D deficiency was generally high (60% for 2014–2017) but dropped by 10% in 2018 and 2019. As such, the summers of 2018 and 2019, which are among the hottest and driest in Germany since the start of modern climate recordings, had a measurable positive effect on 25(OH)D plasma levels of the examined population. Given that 25(OH)D deficiency is widespread in higher latitudes, this implies that while mostly considered negative, climate change might also confer some health benefits with regard to vitamin D related medical conditions.

## Introduction

Climate change has been termed “the biggest global health threat of the 21^st^ century” [1] and is expected to alter the geographic distribution, range and severity of many health risks, like cardiovascular diseases, heat related illness and vector borne diseases [2–4]. Ultimately climate change will likely increase the frequency of extreme weather events like e.g. extended warm spells and heat waves, leading to more areas affected by drought or, on the other extreme, by heavy precipitation events [5]. Also, the exposure of the human population to ultraviolet radiation (UVR) is expected to increase directly due to depletion of the stratospheric ozone layer caused by an interaction with the warming climate system [6, 7]. As a consequence, an increase in the number of warm, sunny days, warm spells and heat waves, caused by climate change, will also indirectly lead to changes in human behaviour with more time being spent outdoors with less protective clothing, again resulting in an increased UVR exposure [7]. While increased UVR exposure has negative effects like increased rates of sun burn and skin cancer, it should also increase vitamin D production, which might be a beneficial effect, at least in higher latitudes where low or insufficient levels of vitamin D are frequent [8–11].

Hypovitaminosis D, or vitamin D deficiency, is commonly defined as a blood 25-hydroxyvitamin D (25(OH)D) concentration of less than 50 nmol/l [12], while 25(OH)D concentrations below 75 nmol/l are considered as insufficient [13–15]. The fundamental biochemical role of vitamin D lies in up-keeping skeletal health, regulating the development and remodelling of bones, with the most prominent manifestation of vitamin D deficiency being rickets [8, 16], which affected more than 25% of children in London less than one century ago and is still a notable threat to children’s health in the developing world [16–18]. Also Osteoporosis, as the main age and bone related condition in developed countries is worsened by vitamin D deficiency [19] and is especially of concern in the aging populations of the highly developed nations. Moreover, many other, non-skeletal disorders, ranging from autoimmune diseases, like multiple sclerosis, over cardiovascular disease up to cancer have been linked to vitamin D deficiency [8, 20–22], even though these findings are currently disputed and low vitamin D status might be of mere correlative nature and could just be a proxy for general poor health [23–25].

As outlined above, and irrespective of its role in non-skeletal health, vitamin D levels in human populations are expected to be influenced by climate change, with increasing temperatures and warm spells resulting in overall elevated 25(OH)D blood concentrations due to increased UVR exposure in affected populations [7]. While this prediction seems plausible and seasonal fluctuations of 25(OH)D levels have been described [11, 26, 27], to our knowledge, no direct observations of such an effect have been reported so far. In this study we tested the hypothesis of increased vitamin D levels due to extreme weather events (which might be already part of global climate change) by using a large clinical data set of 25(OH)D plasma concentrations from in- and out-patients of the University Clinic in Halle (Saale), Germany. The collected data covered the time period from 2014 to 2019, including data from two of the hottest, sunniest and driest summers ever recorded in Germany so far. The specific aims of our study were: 1. Gain insight into the average 25(OH)D blood concentrations and the abundance of 25(OH)D deficiency in a hospital population. 2. To evaluate seasonal patterns in 25(OH)D levels in the data set. 3. To test for changes in the patient population’s average 25(OH)D levels, caused by the weather extremes of 2018 and 2019.

## Methods

### Data collection

This retrospective study was conducted at the University Hospital Halle (UKH), Saxony-Anhalt, Germany. The UKH has approximately 1000 beds and around 40 000 patients are treated on an inpatient basis and 195 000 patients on an outpatient basis per year. All 25(OH)D plasma concentrations were measured and recorded in the Central Laboratory of the UKH and the respective data on 25(OH)D plasma concentrations from 2014 to 2019 were retrieved from the Central Laboratory’s laboratory information system (LIS). Only the first recorded 25(OH)D measurement per patient during the study period was used and all other measurements were excluded from the dataset, to avoid bias due to repeated measurements or effects of potential treatment in the clinic. The 25(OH)D measurements thus included in- and outpatients of the UKH and no exclusion criteria were applied to them. The objective of this study was therefore to focus on an unselected cohort of hospital patients, avoiding a possible selection bias, which is common in most experimental studies. We presume that this is the most conservative, unbiased approach to examine the association between sunshine and vitamin D levels taking advantage of the large sample size available from a clinic laboratory data base.

Meteorological data on monthly sunshine in Saxony-Anhalt from 2014 to 2019 were obtained from the Climate Data Center (CDC-portal) of the German Meteorological Service (Deutscher Wetterdienst—DWD) [28]. This study was approved by the ethics committee of the Medical Faculty of the Martin-Luther-University Halle-Wittenberg (approval 2020–031).

### Laboratory analysis

From January to mid-April 2014 25(OH)D was measured on a Roche E170 modular analytics immunoassay analyser with the Roche Elecsys-Vitamin-D-total assay (ECLIA-electro-chemiluminescence immunoassay, Roche Diagnostics, Mannheim, Germany). From mid-April 2014 to December 2019 25(OH)D was measured with the same assay, but on a Roche cobas e602 analyzer integrated in a fully automated Roche Cobas 8000 platform. Based on a method comparison in accordance with the CLSI EP09-A3 guideline [29], 25(OH)D concentrations measured on the Roche E170 analyser were adjusted, to account for the measurement bias between the analyser systems. All analysis on the Roche E170 and Roche cobas e602 analyser were carried out according to the manufacturer’s instructions and manuals, with routine maintenance and quality control procedures. Besides standard internal quality control schemes according to the Guideline of the German Medical Association on Quality Assurance of Medical Laboratory Examinations (RiliBÄK) [30], the Central Laboratory of the UKH also participates in external quality control schemes for 25(OH)D twice a year with the Reference Institute for Bioanalytics (RfB) [31]. The RfB is one of the two institutions officially designated by the German Medical Association (Bundesärztekammer) for external quality control for medical laboratories. For the period from 2014 to 2019, all 25(OH)D external quality controls were passed successfully.

### Statistical analysis

We used a Kruskall-Wallis one-way ANOVA on ranks (including Dunn’s Post-hoc) to test for differences in 25(OH)D concentrations between the observed years (2014–2019). Further, we tested for a correlation of individual 25(OH)D concentrations and age (Spearman’s rank correlation) and for differences in individual 25(OH)D concentrations between the sexes (Mann-Whitney independent sample test). The above described statistical analyses were carried out with MedCalc Statistical Software (version 19.1.3, Ostend, Belgium) and Microsoft Excel 2016.

To get a nuanced picture of the relation between sunshine and 25(OH)D levels, we used a time series analyses to describe the temporal association of Vitamin D and the amount of sunshine per month. Time series analysis, and more specifically SARIMAX (seasonal autoregressive integrated moving average) models, were used as statistical analyses. Such models contain a moving average and an autoregressive term. While the former models the impact of random fluctuations on the expected mean, the latter estimates the association of a value at a certain time point with its predecessor. The order, that is number of such terms, is given in brackets as (p = autoregressive, d = grad of differentiation, q = moving average). Model identification was based on the algorithm introduced by Box and Jenkins [32, 33] which is described in detail in the supplement together with further details of model identification and parameter estimation (S1 Appendix). Results from the different SARIMAX models are given as the additional 25(OH)D levels per hour of sunshine, together with their corresponding 95% confidence intervals. The limit of significance was assumed to be α = 0.05. Additionally, models were adjusted for the mean age and the proportion of female cases in every month. To model the time series we used the R-Function ‘arimax’. A sinus curve was fitted to the data of monthly sunshine in order to illustrate the average seasonal fluctuation across the observational period. Time series analyses and data management were performed using R (Version 3.6.0) [34].

### Sensitivity analysis

The very low zenith angle of the sun during winter is considered to be a potential cause for the complete lack of availability of UV radiation for 25(OH)D production [35–37]. In order to account for a potential interruption of 25(OH)D production during winter, which might not be reflected by sunshine hours, we performed a sensitivity analysis where we excluded the winter months with the lowest sun altitude (January, November and December). Comparing the correlation between 25(OH)D and sunshine hours with the full data set, we computed the Spearman correlation coefficient for each of the two approaches.

## Results

**Fig 1:**
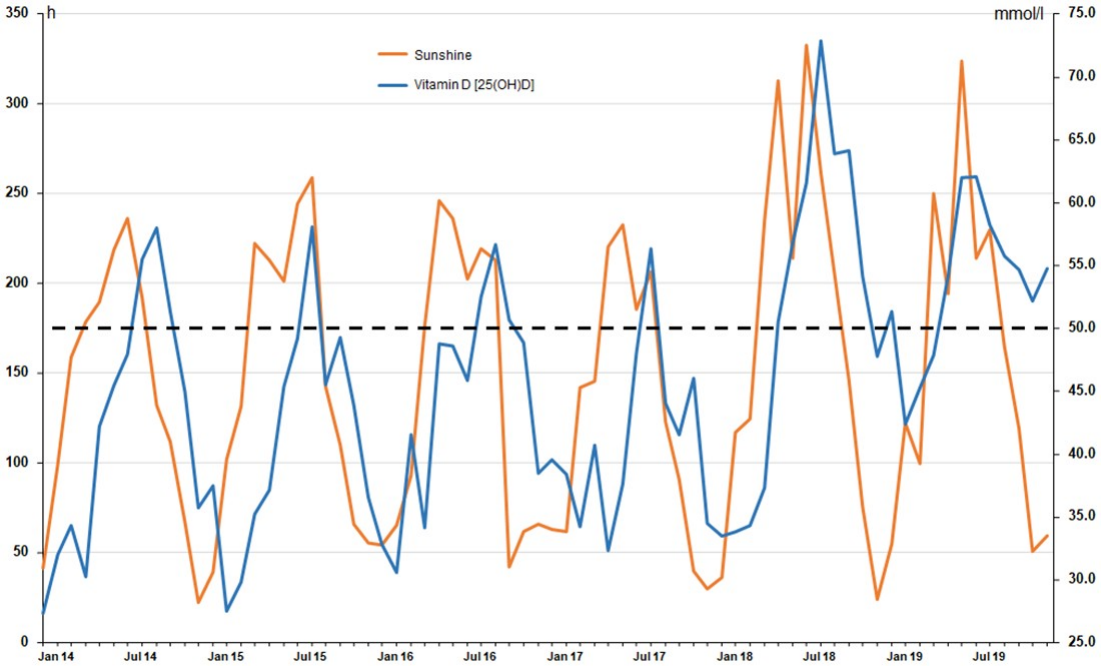
Sunshine and median 25(OH)D levels. Hours of monthly sunshine (left x-axis; orange line) and the monthly median 25(OH)D concentrations in nmol/l (right x-axis; blue line) of the study population for the time period from January 2014 to December 2019.

A total of 13 406 individual Vitamin D measurements were retrieved from the LIS of the central laboratory and were used in the analysis. Table 1 gives a summary of sample size and average age of the population per year, the hours of sunshine accumulated per year, as well as the median vitamin D 25(OH)D concentrations and the percentage of Vitamin D deficient or insufficient patients. Fig 1 depicts the median monthly 25(OH)D concentrations and the monthly sunshine for the study period. The monthly median 25(OH)D levels for 2104–2019, their sample sizes and their 25^th^ to 75^th^ percentiles are presented in detail in S1 Table.

**Table 1:** Study population characteristics, 25(OH) D levels and sunshine.

year	n	f	m	age (SD)	sunshine	25(OH)D (25^th^– 75^th^)	<25 nmol/l	<50 nmol/l	<75nmol/l	>125 nmol/l
2014	1626	988	638	56.1 (17.3)	1648	41.6 (23.6–63.5)	26.8	61.1	83.0	2.3
2015	1700	1032	668	56.4 (16.6)	1787	40.7 (21.9–67.3)	29.2	60.4	81.2	2.8
2016	1703	972	731	58.1 (16.5)	1676	43.2 (16.5–69.9)	27.4	56.9	78.8	4.1
2017	2219	1243	976	60.8 (18.4)	1542	41.1 (22.3–66.3)	28.7	60.4	80.4	4.2
2018	2903	1597	1306	62.3 (17.8)	2086	50.8 (29.9–79.2)	19.6	48.8	71.5	6.7
2019	3255	1843	1412	62.1 (18.3)	1882	52.8 (30.2–80.3)	18.7	47.0	71.2	6.8

Total sample sizes (n), number of women (f), number of men (m), the average age in years and the corresponding standard deviation (SD), the amount of sunshine per year in hours and the median 25(OH)D concentrations as well as the corresponding 25th to 75th percentile are given for the years 2014 to 2019. The percentage of patients in the population with 25(OH)D blood concentrations < 25 nmol/l (severely deficient), < 50nmol/l (deficient), < 75nmol/l (insufficient) and > 125 nmol/l (risk associated) are also presented.

From 2014 to 2017 the median 25(OH)D concentrations were below the threshold Vitamin D deficiency (50 nmol/l) for 9–11 months per year, whereas 2018 only 5 and 2019 only 3 months had median 25(OH)D levels below this threshold. Overall, from 2014 to 2017 approximately 60% of the population were vitamin D deficient and 81% vitamin D insufficient (below 75 nmol/l 25(OH)D), while these percentages dropped to 48% for deficiency and to 71% for insufficiency from 2018 to 2019. A congruent trend can also be observed for the percentage of the population affected by severe vitamin D deficiency (<25 nmol/l 25(OH)D), which declined from approximately 28% for the 2014–2017 period to 19% for the years 2018/19. Also concerning patients with vitamin D levels exceeding 125 nmol/l, the years 2018/19 showed a small, but nevertheless higher proportion than to the four previous years (Table 1).

The Kruskall-Wallis analysis revealed that the 25(OH)D concentrations differed significantly between the study years (p <0.001). Based on that, the Post-hoc analysis (Dunn, p<0.001) further revealed that the two record-summer years of 2018 and 2019, while not differing from each other, had significantly higher 25(OH)D concentrations than the previous four years (2014–2017), which however did not differ from each other. The details of the Kruskall-Wallis and Post-hoc analysis are given in S2 Table. Overall 25(OH)D concentrations were negatively correlated with patient age (Spearman’s ρ = -0.052; p <0.0001) and women had significantly higher 25-H concentrations than men (median women = 47.9 mmol/l; median men = 44.3 mmol/l; p < 0.0001).

### Time series analyses

Following the Box-Jenkins approach, we identified a ARIMA (1,0,1) model to describe the time series according to the predefined criteria. Details of the model are given in detail for plots of the autoregression and partial autoregression (S1 and S2 Figs). In the time series analysis, also considering a transfer function, we found that the 25(OH)D levels rose by 0.04 nmol/l (95% CI: 0.04–0.05 nmol/l) per hour of sun shine. The effect estimates changed little when the model was adjusted for the mean age and the proportion of female cases to 0.04 nmol/l 25(OH)D (95% CI: 0.04–0.05 nmol/l) per hour of sunshine. The autoregressive term was close to one in all cases indicating a persisting effect of sun shine on Vitamin D levels in the population (crude model: φ = 0.79, adjusted model: φ = 0.79). From the fit of a sinus curve we found that the summer month in 2018 and 2019 showed an especially high amount of sun shine exceeding the average long-term fit.

### Sensitivity analysis

In the sensitivity analysis, excluding all winter months with very low sun altitude (November to January), we found an even stronger correlation between sunshine hours and 25(OH)D (Spearman correlation = 0.68), as compared to the full data set (Spearman correlation = 0.57). As such, the statistical approach used in this study is indeed a feasible, conservative model which does not overestimate the impact of sunshine on vitamin D production.

## Discussion

In this study we retrospectively analyzed 25(OH)D levels of a large hospital population, covering the years 2014 to 2019, thus including two of the hottest summers ever recorded in Germany. Overall, our analyses revealed, that in all years a high percentage of patients (51% +/- 6.2%) were in fact vitamin D deficient [19], with 25(OH)D blood concentrations below 50 nmol/l (Table 1). When applying the wider definition of vitamin D insufficiency (25(OH)D concentrations of < 75 mmol/l), the proportion of affected patients even rises to more than three quarters (78% +/- 5.1%). However, as can be seen from Fig 1, the monthly median 25(OH)D blood levels vary considerably following the accumulated hours of sunshine per month and as such, vitamin D deficiency is not equally distributed over the year. This strong seasonal 25(OH)D variation described in our study is congruent with the results of many previous studies [26, 27, 38–40], where the highest 25(OH)D levels occurred during August and September and the lowest are found from January to March [27, 41]. The main difference to previous studies however is our dataset, which is based on a hospital population covering six years, rather than the typical, supposedly healthy population with a more limited sample size, more commonly used for epidemiological studies. The primary objective of this study was thus to estimate the total effect of sunshine on 25(OH)D levels. For the analyses to yield correct effect estimates we however abstained from adjusting for direct or indirect effects of sunshine (such as clothing or medication) on 25(OH)D levels. As sunshine is an independent variable and no individual human action can effect sunshine, no confounders can bias the total effect. For example, light clothing (which increases UV exposure) is caused by sunshine and not the other way round.

Nevertheless, despite our data set being based on a clearly diseased and elderly population (in- and out-patients, no exclusion criteria applied), the seasonal pattern of 25(OH)D concentrations followed the recorded sunshine in a near perfect manner, with the adjusted autoregression of the time series analysis being φ = 0.7 and 25(OH)D levels rising by 0.04 nmol/l (95% CI: 0.04–0.05 nmol/l) per additional hour of sunshine. The gradual increase in 25(OH)D after sun exposure has been estimated to reach its maximum after 7 to 14 days [41, 42], with an even longer 25(OH)D half-life of 25 days [41, 43], which is also reflected in the approximately one month delay of 25(OH)D production and decline in relation to sunshine present in our data (Fig 1).

For both 2018 and 2019, which had the most hours of sunshine during the study period, significantly higher 25(OH)D concentrations occurred than in the previous four years (p<0.001, S2 Table), with the median 25(OH)D concentrations increasing by approx. 10 nmol/l, which reduced the percentage of vitamin D deficient patients by 10–12% (Table 1). Thus, the record summers of 2018 and 2019 had a measurable positive effect on the vitamin D status of a large hospital population, despite the fact, that these persons are expected to be less active and less exposed to sunshine than the rest of the supposedly healthier population. Interestingly, also the small percentage of patients with 25(OH)D concentrations above 125 nmol/l seems to be increased in 2018/19 (mean: 6,7%) when compared to the four previous years (3,4%). While acute toxicity of 25(OH)D is defined by concentrations >375 nmol/l [44], the 2011 report on dietary requirements for calcium and vitamin D from the Institute of Medicine (IOM) recommended to consider chronic 25(OH)D concentrations of >125 nmol/l as risk associated [45, 46]. However given the strong seasonal pattern it seems unlikely that such high concentrations are sustained over the course of the whole year.

So what are the implications of this measurable reduction in hypovitaminosis D and generally increased vitamin D levels due to extreme warm summers? Since climate change is expected to increase the frequency of summer extremes [6, 7], our study supports the hypothesis, that populations living in higher latitudes should be affected by an overall improvement of vitamin D status [7]. To which extend this will be one of the few positive effects of climate change on global human health, ultimately depends on whether vitamin D indeed plays a fundamental role in various non skeletal diseases [9, 23], which is currently a topic of debate within various medical sub-disciplines [20, 47]. Also, other long term climate factors might impact sunshine exposure and UV-radiation, as indicated by the rickets incidence rates in the UK, declining from the 1960’s to mid-1990’s to then drastically increasing again, which has been attributed to Atlantic Multidecadal Oscillation (AMO), a global climate phenomenon with oscillation periods of up to 80 years [10].

Irrespective of the health effects of vitamin D levels, the analysis of 25(OH)D blood concentrations in our study proved to be a sensitive tool reflecting the effects of even short time changes in climate pattern on human physiology. Future combined analyses of larger supra-regional 25(OH)D data sets could provide more detailed insights, also into other factors influencing vitamin D status, but to date such approaches are still hampered by the variability and lack of standardization of vitamin D assays [48].

## Supporting information

S1 Table Median monthly 25(OH)D levels (nmol/l) for 2104–2019, their sample sizes and 25th to 75th percentiles. (XLSX)

S2 Table Kruskall-Wallis and Post-hoc (Dunn) analysis of 25(OH)D concentrations (nmol/l) measured from 2014 to 2019. (DOCX)

S1 Appendix Time series analysis details. (DOCX)

S1 Fig Monthly hours of sunshine. Blue line: Fitted sinus function.
(TIF)

S2 Fig Plot of autocorrelation and residuals. ACF: autocorrelation function.
(TIF)
