# Supplementary material for: Do extreme summers increase blood vitamin D (25-hydroxyvitamin D) levels?
Source: PLoS One. 2020 Nov 10;15(11):e0242230. doi: 10.1371/journal.pone.0242230 (PMC7654803; doi:10.1371/journal.pone.0242230)
Supplement: S2 Table — (DOCX) [file pone.0242230.s002.docx]

| **Kruskal-Wallis test** | | | | |
| --- | --- | --- | --- | --- |
| Test statistic | | | 289.03 | |
| Corrected for ties  Ht | | | 289.04 | |
| Degrees of Freedom (DF) | | | 5 | |
| Significance level | | | P<0.001 | |
| **Post-hoc analysis (Dunn)** | | | | |
| Factor | n | Average Rank | | Different (P<0.001) from factor |
| 2014 (1) | 1626 | 6102.2 | | (5)(6) |
| 2015 (2) | 1700 | 6105.3 | | (5)(6) |
| 2016 (3) | 1703 | 6374.2 | | (5)(6) |
| 2017 (4) | 2219 | 6166,7 | | (5)(6) |
| 2018 (5) | 2903 | 7238.5 | | (1)(2)(3)(4) |
| 2019 (6) | 3255 | 7377.4 | | (1)(2)(3)(4) |
