# Supplementary material for: Do extreme summers increase blood vitamin D (25-hydroxyvitamin D) levels?
Source: PLoS One. 2020 Nov 10;15(11):e0242230. doi: 10.1371/journal.pone.0242230 (PMC7654803; doi:10.1371/journal.pone.0242230)
Supplement: S1 Appendix — (DOCX) [file pone.0242230.s003.docx]

**S1 Appendix**

As the methods used in our analysis are rarely applied in medical research, we provide a short overview of the essentials in this appendix, a comprehensive account can be found in Helfenstein [1]. We performed a so called intervention analysis, which is a special case of a transfer function analysis. The basic idea beyond a transfer function is to display a time series y_t_ (number of deaths per day in our case) as the sum of a noise series n_t_ and a transfer series u_t ._

| $y_{t}=n_{t}+u_{t}$ |
| --- |

The noise series n_t_ is the part of the observed time series that cannot be explained by the effect of the input series and captures all of the typical features of a time series such as trends, seasonality, or autocorrelation. In an intervention analysis the transfer series u_t_ consists of a input series I_t_, which, in our case, is the time series of hours of sunshine in Saxony-Anhalt w_0_.

| $u_{t}=w_{0}\cdot I_{t}$ |
| --- |

To give an example, when there is one hour sunshine the formula take the form $y_{t}=n_{t}+w_{0}\cdot1=n_{t}+w_{t}$. Thus, the pulse w_0_ is simply the excess of blood concentration of Vitamin D due to one additional hour of sunshine.

The noise series n_t_ can in most cases (and also in our work) be adequately modelled by a SARIMA-model (seasonal ARIMA model), which is expressed by ARIMA(p,d,q)(P,D,Q)_s_. To better understand this expression, let us first consider an ARIMA(p,d,q) model and leave the (P,D,Q)_s_ part for later discussion. Basically, an ARIMA model consists of three terms which model the order of the AR (autoregressive, p), the I (integrated, d), and the MA (moving average, q) part of n_t_.

**(1) The AR part:**

For convenience, we start with an AR(1) model, that is, an AR model with p=1. This can be interpreted like an ordinary linear regression equation with y_t_ as the response, φ as the regression parameter for the single covariate y_t-1,_ and a normally distributed random error e_t_.

$$y_{t}=\phi y_{t-1}+\epsilon_{t}$$

The current value of the model, y_t_ is the sum of the previous value y_t-1_ (multiplied by φ) and the random error. The association between y_t_ and y_t-1_ is controlled by the AR(1) parameter φ: the larger φ, the higher is the association between y_t_ and y_t-1_. The idea of regressing the current value on its own predecessor explains the term autoregressive for this model. Autoregressive models of higher orders (AR(p) models) are straightforward extensions of the AR(1) process, including the p previous values of the process in the model equation.

| $y_{t}={\phi_{1}y}_{t-1}+{\phi_{2}y}_{t-2} +\ldots+{\phi_{p}y}_{t-p}+\epsilon_{t}$ |
| --- |

When the value of $\phi$ equals one the current value is expected to have the same value as its predecessor resulting in a constant time series. When adapted to the transfer function, a value of the autoregressive term close to one indicates likewise a persistent effect. In this case the input series $I_{t}$ itself is a time series incorporating an autoregressive term.

**(2) The MA part:**

The idea of an MA model is similar to that of an AR model; however, now the current value of the time series, y_t_, is assumed to depend only on random fluctuations. If random fluctuations on the same day (ε_t_), and on the day before (ε_t-1_) are taken into account, a MA(1) model is defined.

| $y_{t}=\epsilon_{t}+\theta\epsilon_{t-1}$ |
| --- |

Higher orders q of an MA model are straightforwardly defined as

| $y_{t}=\epsilon_{t}+\theta\epsilon_{t-1}+...+\theta_{q}\epsilon_{t-q}$ |
| --- |

**(3) The I part**

A time series is said to be stationary, if the mean of the time series does not depend upon time, but is constant throughout the complete time course. For a valid intervention analysis, the noise series n_t_ has to be stationary. The easiest way to achieve stationarity is by differentiating the time series by the preceding value s time points ago, where s is the length of the period. Further differentiations with different lags are possible (with d measuring the number of differentiations), but were not necessary in our case.

In the SARIMA model, the seasonal aspect of the noise series n_t_ is additionally (to the former ARIMA(p,d,q) part) expressed by an ARIMA(P,D,Q)_s_ term. This seasonal term is assumed to be another ARIMA model with own orders P, D, Q, a seasonal lag parameter s, and own model parameters Φ and Ω (now written as capital letters).

Actual model fitting thus involves finding the optimal orders of the SARIMA model, the respective parameters, and the parameter of actual interest, the pulse w_0_. Box/Jenkins [2] proposed an algorithm for this model identification which is frequently used in applied research. This algorithm consists of four steps:

**(1) Make the original time series (y_t_) stationary**

Stationarity can be checked via the Dickey-Fuller test. An underlying trend or seasonality is assessable via the empirical autocorrelation and partial autocorrelation functions of y_t_ at various lags. The autocorrelation at lag k is the correlation of the value y_t_ and its predecessors y_t-k_. The partial autocorrelation at lag k adjusts for the influence of time points lying between the value y_t_ and its predecessor y_t-k_, leaving only the adjusted correlation between the two values.

In our case, we found a seasonality of seven days in all 15 models, thus SARIMA models that were differentiated with a lag of seven were fitted (ARIMA(p,0,q)(P,1,Q)_7_).

**(2) Find a preliminary order of the model**

In the second step, a preliminary order of the ARIMA(p,0,q)(P,1,Q)_7_ model (that is, p,q, P, and Q) is identified by again referring to the autocorrelation functions.

**(3) Estimate the coefficients of the model**

The coefficients θ, φ, Φ and Ω are estimated by maximum likelihood.

**(4) Check the model by assessing the autocorrelations of the residuals**

As a final step, the adequacy of the model from step (3) has to be evaluated, which is done by demanding no relevant autocorrelations of the residuals. If there are no autocorrelations, the model can be regarded as properly modeling the noise series n_t_. Finally, all coefficients (θ, φ, Φ and Ω) from the noise series are estimated again by maximum likelihood, but now simultaneously with the pulse w_0_. Here, Rinne/Specht [3] suggest a maximum lag of $K\approx2\sqrt{T}$ (in our case the lag length is about 140) when the residuals are checked for autocorrelations as implemented in the method described by Box/Jenkins. Therefore, a lag of 365 days is out of the range to be considered.

**References**

1. Helfenstein U. Box-Jenkins modelling in medical research. Stat Methods Med Res. 1996;5:3-22.
2. Box GEP JGM (1976): Time series analysis: Forecasting and control. San Francisco: Holden-Day [Holden-Day series in time series analysis and digital processing].
3. Rinne H, Specht K Zeitreihen: Statistische Modellierung, Schätzung und Prognose [Time Series: Statistical Modelling, Estimation, Prognosis]. Munich: Vahlen, 2002.
