# Supplementary figures and images for: Do extreme summers increase blood vitamin D (25-hydroxyvitamin D) levels?
Source: PLoS One. 2020 Nov 10;15(11):e0242230. doi: 10.1371/journal.pone.0242230 (PMC7654803; doi:10.1371/journal.pone.0242230)

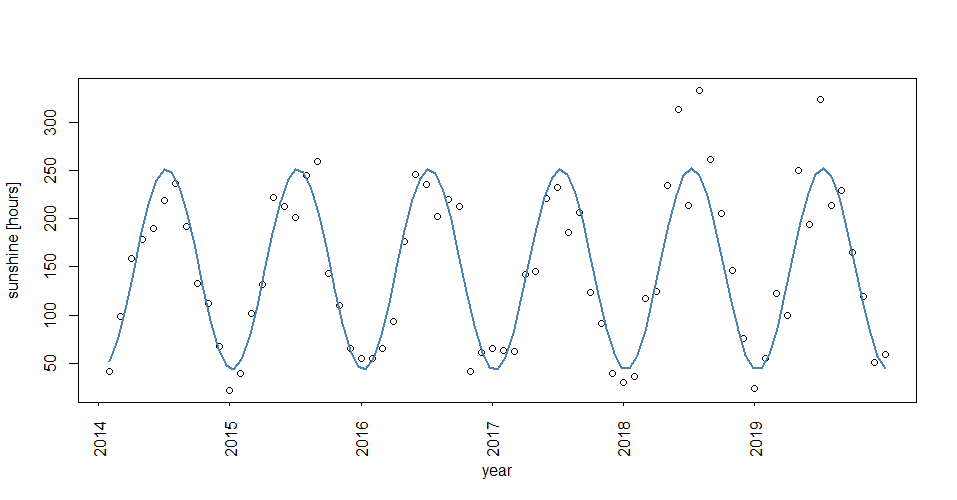

Supplement: S1 Fig — Blue line: Fitted sinus function. (TIF) [file pone.0242230.s004.tif]

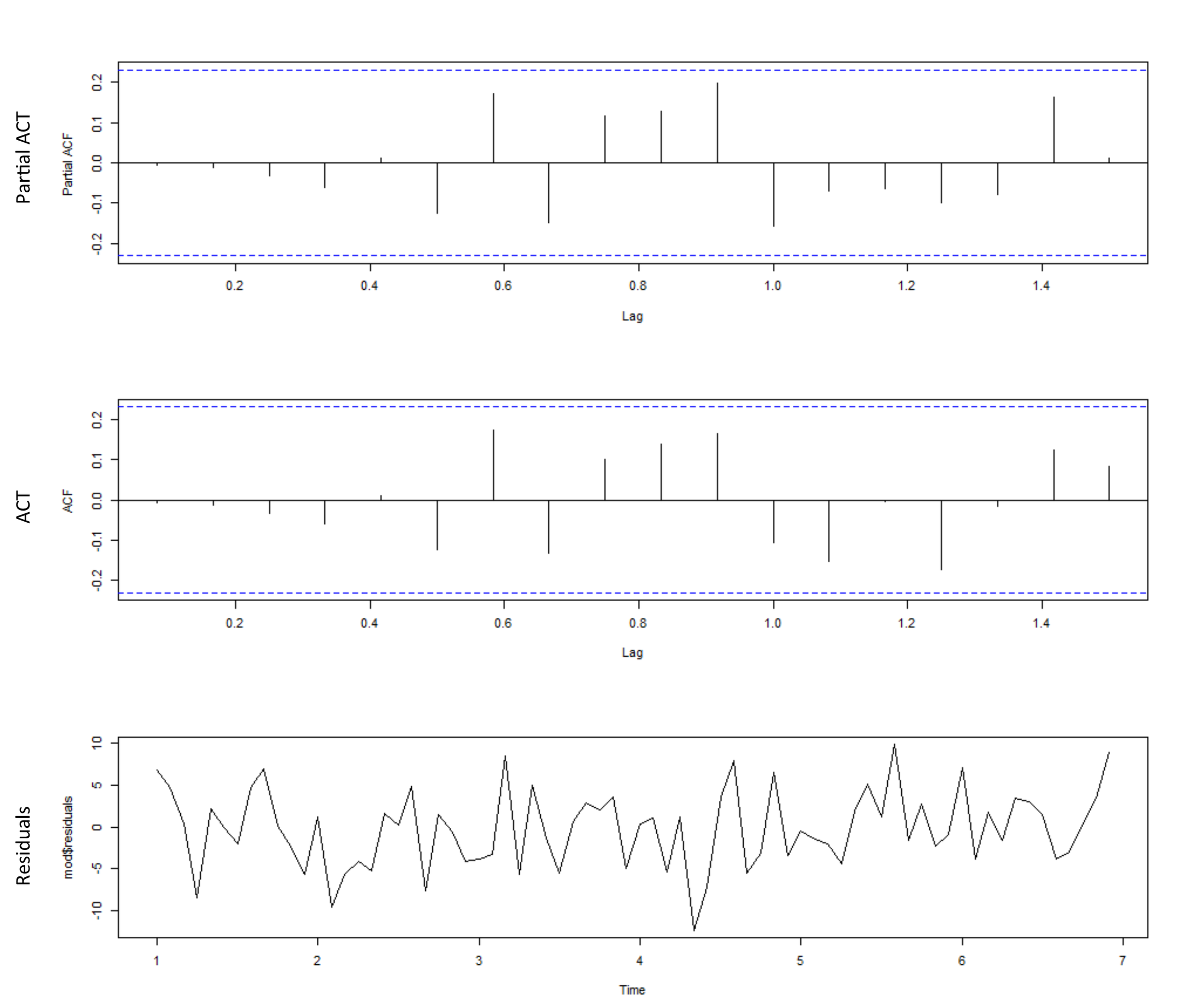

Supplement: S2 Fig — ACF: autocorrelation function. (TIF) [file pone.0242230.s005.tif]
